# [Expression of Concern] ISL1 promotes cancer progression and inhibits cisplatin sensitivity in triple-negative breast cancer cells

**DOI:** 10.3892/ijmm.2026.5846

**Published:** 2026-05-06

**Authors:** Yang Zhang, Lu Wang, Peng Gao, Zhiguo Sun, Ning Li, Yanqin Lu, Jianglun Shen, Jian Sun, Yiming Yang, Hao Dai, Haifeng Cai

Int J Mol Med 42: 2343-2352, 2018; DOI: 10.3892/ijmm.2018.3842

Following the publication of this paper, it was drawn to the Editor's attention by a concerned reader that, regarding the cell invasion assay experiments shown in Fig. 3A, B and C on p. 2348, two pairs of data panels were overlapping, such that data which were intended to show the results from differently performed experiments had apparently been derived from a smaller number of original sources. In addition, an independent investigation of the data in this paper undertaken by the Editorial Office revealed that flow cytometric data in Fig. 4A were shared with a paper that was published in the journal *OncoTargets and Therapy* featuring no authors in common (which was received at that journal one month after the submission of the above paper to *International Journal of Molecular Medicine*), and flow cytometric data in Fig. 5C were shared with a different paper also having no authors in common that was subsequently submitted to the journal *Oncology Reports*.

The authors have been contacted by the Editorial Office to offer an explanation for the issues described above, and we are awaiting their response. Owing to the fact that the Editorial Office has been made aware of potential issues surrounding the scientific integrity of this paper, we are issuing an Expression of Concern to notify readers of this potential problem while the Editorial Office continues to investigate this matter further.

